# Exercise Training Effects on Circulating Endothelial and Progenitor Cells in Heart Failure

**DOI:** 10.3390/jcdd9070222

**Published:** 2022-07-10

**Authors:** Christos Kourek, Alexandros Briasoulis, Virginia Zouganeli, Eleftherios Karatzanos, Serafim Nanas, Stavros Dimopoulos

**Affiliations:** 1 Clinical Ergospirometry, Exercise & Rehabilitation Laboratory, 1st Critical Care Medicine Department, Evangelismos Hospital, National and Kapodistrian University of Athens, 10676 Athens, Greece; chris.kourek.92@gmail.com (C.K.); lkaratzanos@gmail.com (E.K.); sernanas@gmail.com (S.N.); 2 Department of Cardiology, 417 Army Share Fund Hospital of Athens (NIMTS), 11521 Athens, Greece; 3 Department of Clinical Therapeutics, Alexandra Hospital, Faculty of Medicine, National and Kapodistrian University of Athens, 11528 Athens, Greece; alexbriasoulis@gmail.com; 4 Division of Cardiovascular Medicine, Section of Heart Failure and Transplantation, University of Iowa Hospitals and Clinics, Iowa City, IA 52242, USA; 5 Second Cardiology Department, Attikon University Hospital, Medical School, National and Kapodistrian University of Athens, 12462 Athens, Greece; virginia_noa@yahoo.gr; 6 Cardiac Surgery Intensive Care Unit, Onassis Cardiac Surgery Center, 17674 Athens, Greece

**Keywords:** heart failure, exercise training, acute exercise, endothelial progenitor cells, circulating endothelial cells, endothelium

## Abstract

Heart failure (HF) is a major public health issue worldwide with increased prevalence and a high number of hospitalizations. Patients with chronic HF and either reduced ejection fraction (HFrEF) or mildly reduced ejection fraction (HFmrEF) present vascular endothelial dysfunction and significantly decreased circulating levels of endothelial progenitor cells (EPCs). EPCs are bone marrow-derived cells involved in endothelium regeneration, homeostasis, and neovascularization. One of the unsolved issues in the field of EPCs is the lack of an established method of identification. The most widely approved method is the use of monoclonal antibodies and fluorescence-activated cell sorting (FACS) analysis via flow cytometry. The most frequently used markers are CD34, VEGFR-2, CD45, CD31, CD144, and CD146. Exercise training has demonstrated beneficial effects on EPCs by increasing their number in peripheral circulation and improving their functional capacities in patients with HFrEF or HFmrEF. There are two potential mechanisms of EPCs mobilization: shear stress and the hypoxic/ischemic stimulus. The combination of both leads to the release of EPCs in circulation promoting their repairment properties on the vascular endothelium barrier. EPCs are important therapeutic targets and one of the most promising fields in heart failure and, therefore, individualized exercise training programs should be developed in rehabilitation centers.

## 1. Introduction

Heart failure (HF) presents a major public health issue worldwide with a tremendous burden on healthcare systems and their resources due to the high number of hospitalizations and readmissions among diagnosed adults and the elderly [1,2]. It is estimated that approximately 20% of HF patients are readmitted in the US hospitals within the first 30 days, while the respective number in several European countries is lower [2,3,4]. The annual incidence of HF presents a linear increase with age and ranges widely from 1 to 9 cases per 1000 person-years in both Europe and the US, with the median number rising to 3.20 (IQR 2.66–4.17) cases [4,5]. The median length of stay is 8.50 (IQR 7.38–10) days [4]. Moreover, the prevalence of HF according to the 2021 American Heart Association Statistical Update is estimated between 1.5% and 1.9% of the total US and Canadian population, while in Europe it ranges between 1% and 2% [4,6,7].

Patients with HF usually present with impaired endothelium-dependent vasodilation, endothelial nitric oxide synthases (eNOS) uncoupling, and reduced availability of nitric oxide (NO) [8,9,10]. Vascular endothelial dysfunction caused by increased formation of superoxide radicals and other oxidant species, and “oxidative stress” result in reduced exercise capacity and, thus, in worse quality of life [8,9,10]. Exercise has been shown to have beneficial effects in vasodilation and, therefore, endothelial function resulting in higher exercise capacity and better quality of life between HF patients [11,12,13,14]. In addition, exercise is a strong recommendation (Class IA) of treatment in heart failure according to the latest ESC [15,16] and AHA Guidelines [17,18].

Endothelial progenitor cells (EPCs) are bone marrow-derived cells involved in endothelium regeneration, homeostasis, and neovascularization [19,20]. They either transform in mature circulating endothelial cells (CECs) or remain as precursor cells restoring the dysfunctional and injured endothelium and promoting vasculogenesis and angiogenesis [19,20]. Recent studies have shown that circulating levels of EPCs and CECs are significantly decreased in HF patients with vascular endothelial dysfunction and inflammation compared to age-matched healthy subjects without established cardiovascular disease [21]. Thus, CECs and EPCs could be suggested as potential biomarkers of the cellular response to vascular injury in patients with HF [21]. Although exercise has been suggested to have a positive impact on the mobilization of EPCs and the increase of CECs in cardiovascular diseases (especially HF), our knowledge still remains limited regarding this field. There is also confusion in recognizing and defining different progenitor cell populations.

The aim of the present review is to demonstrate the most updated knowledge regarding the acute and long-term effects of exercise on EPCs and CECs in patients with chronic HF and either reduced ejection fraction (HFrEF) or mildly reduced ejection fraction (HFmrEF) and present, in a more clarified way, the most approved methods of identification.

## 2. Circulating Endothelial and Progenitor Cells

### 2.1. Definition and Identification

Endothelial progenitor cells (EPCs) are bone marrow-derived cells contributing to the shielding of vascular protection, the restoring of dysfunctional and injured endothelium, the promotion of angiogenesis, and the regulation of vascular homeostasis [19,20]. There are numerous and complex proposed signaling pathways in the EPCs mobilization and differentiation into mature endothelial cells. Although EPCs are usually isolated from the bone marrow or peripheral blood, there are cases where they have been isolated from fetal liver or umbilical cord blood [22,23].

An important issue in the field of EPCs is the lack of an established method for their identification in the human system. The most widely approved method of EPCs identification is the use of monoclonal antibodies and fluorescence-activated cell sorting (FACS) analysis via flow cytometry in order to quantify specific cell populations. Agreement between methods for EPC quantification is moderate to poor, which may explain controversies in the literature [24]. The first investigators that referred to EPCs were Asahara T. et al. [25] back in 1997. More specifically, Asahara T. et al. described EPCs as a population of peripheral blood mononuclear cells which express the hematopoietic stem marker CD34 and another endothelial cell marker, known as vascular endothelial growth factor receptor 2 (VEGFR-2) or kinase insert domain receptor (KDR) or Flk-1 [1]. This population of peripheral blood mononuclear cells have the possibility to differentiate into circulating endothelial cells [25]. Since this description, many investigators have proposed methods of identification and quantification of EPCs from other blood cell elements through different phenotype protocols in cardiovascular diseases and cancer [26,27,28,29,30,31,32,33,34,35,36]. The common element among these protocols is that EPCs expressed the monoclonal antibodies CD34 and VEGFR-2.

**Figure 1:**
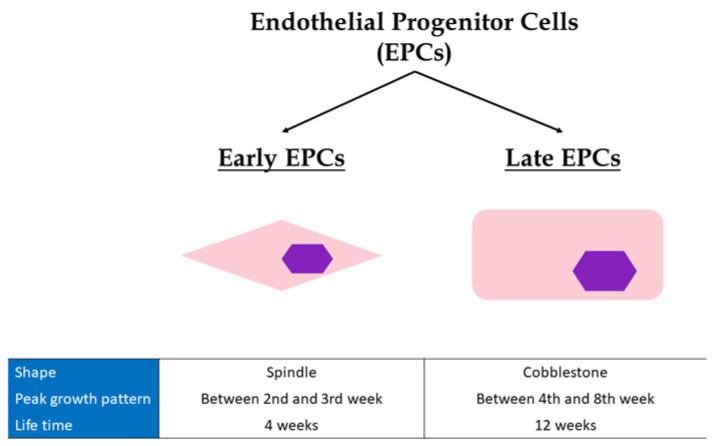
Differences between early and late Endothelial Progenitor cells. EPCs, endothelial progenitor cells.

EPCs are not a single type of cell population. Actually, there are two main groups: the precursor endothelial cells from the bone marrow, known as “early EPCs”, and the more mature endothelial cells, known as “late EPCs” [37]. There are main differences between these two populations in their shape, peak growth pattern, and mean lifetime (Figure 1). Most specifically, early EPCs present a spindle shape while late EPCs a cobblestone shape [37]. The peak growth of early EPCs is between the 2nd and 3rd week while late EPCs have exponential growth between the 4th and 8th week. Finally, early EPCs have a mean lifetime of 4 weeks, whereas the respective mean lifetime of late EPCs is 12 weeks [37]. Late EPCs have the capacity to produce more NO, incorporate faster and more easily into human umbilical vein endothelial cells monolayer, and restore vascular wall better than early EPCs, behaving functionally more like mature endothelial cells (CECs) [37]. However, they have a much higher proliferation rate, and longer survival than CECs. Late EPCs secrete less angiogenic factors compared to early EPCs [38,39]. Nevertheless, both show comparable in vivo vasculogenic capacity [37].

Another cell surface glycoprotein, CD133, has been also used as an early hematopoietic stem-cell marker the last years and identifies hematopoietic stem and progenitor cells from human bone marrow, fetal liver, and peripheral blood [40]. CD133 is not detectable on the surface of human vein CECs [40]. Thus, expression of monoclonal antibodies CD34, VEGFR-2, and CD133 and, therefore, a possible phenotype of CD34+/VEGFR-2+/CD133+ cells could distinguish subgroups localized predominantly in the bone marrow [41]. This phenotype of cells does not express markers such as vascular endothelial (VE) cadherin and von Willebrand factor [40,41]. On the other hand, although more mature EPCs which have been differentiated into CECs in the peripheral circulation of adults present a high expression of CD34 and VEGFR-2, they seem to have lost the expression of CD133. Moreover, compared to EPCs, CECs are shown to express VE-cadherin and von Willebrand factor [40,41,42]. Late EPCs have a similar profile expression to CECs (CD34+, VE-Cad+, vWF+) but, in contrary to CECs, they express CD133. Conclusively, the loss of CD133 expression, either during the transmigration of the immature EPCs from the bone marrow into the systemic circulation or later during their circulation, reflects their transformation into mature CECs [42]. Taking all these factors into consideration, we could extract a safe hypothesis that the expression of CD133 could be a clear point of discrimination between EPCs and CECs and that EPCs population could be identified using CD34 or CD133 or VEGFR-2 (KDR) or any combination of them [43].

Compared to EPCs which are positive for CD45, CECs are identified as CD34+/VEGFR-2+ cells that are negative for CD45 [43,44]. The common denominator of identifying CECs is the CD34+high/VEGFR-2+/CD45- profile [44]. The relationship between EPCs and hematopoietic stem and progenitor cells is still under investigation [45]. EPC populations have been shown to co-express hematopoietic markers including CD34, CD133, CD105, and CD117. Moreover, studies have shown that additional markers such as CD31, CD144, and CD146 could also be expressed by EPCs, either as substitutes for VEGFR2 or to further refine the population [44,46,47,48]. Other markers which are being examined in order to identify tissue-resident endothelial stem cells, especially in mice, are CD157 and endothelial protein C receptor (EPCR) [49,50]. Thus, the most frequently used markers, so far, are CD34, VEGFR-2, CD31, CD144, and CD146. Among them, CD34 alone appears as the top cell marker of identification, while the combination between CD34 and VEGFR-2 is the second most common identity [51].

The major problem in the identification of endothelial cell populations is the fact that all of these surface markers mentioned above are not specific for the identification of EPCs and CECs. Monoclonal antibodies could be used to identify other cellular populations such as dendritic cells, lymphocytes, and macrophages. More specifically, VEGFR-2 could also be expressed in dendritic cells, macrophages, and T lymphocytes while CD34 in various mature endothelial cells [52]. CD31 could be expressed in mature endothelial cells, CD45 neutrophils, monocytes, dendritic cells, and lymphocytes, while CD105 and CD117 are expressed on hematopoietic stem cells, and CD146 on memory lymphocytes [45,53,54,55,56]. 

**Figure 2:**
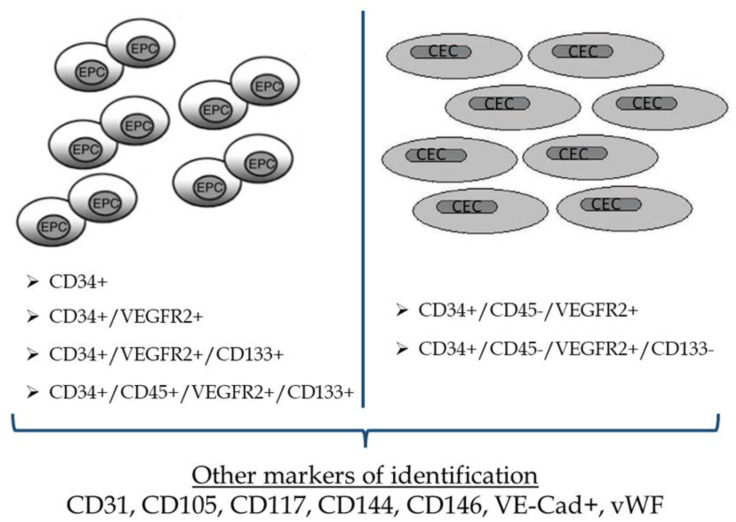
Most common markers used for the identification of Endothelial Progenitor cells and Circulating Endothelial cells. EPCs, endothelial progenitor cells; CECs, circulating endothelial cells; VEGFR, vascular endothelial growth factor receptor.

Based on the current knowledge, EPCs may be identified as CD34+ or CD34+/VEGFR-2+ or CD34+/VEGFR-2+/CD133+ or CD34+/CD45+/VEGFR-2+/CD133+, while CECs could be identified as CD34+/CD45-/VEGFR-2+ or CD34+/CD45-/VEGFR-2+/CD133-. The addition of other surface markers in these established identification profiles could be very helpful but is still under investigation (Figure 2).

EPCs identification is a controversial field of discussion due to the lack of an established method of identification and specific guidelines on this procedure. The use of monoclonal antibodies through flow cytometry is an accurate but high-cost procedure, with still limited knowledge regarding its prognostic value compared to other cheaper markers in HF. If experts manage to establish a common method of EPCs identification and explain the role and the function of each cellular population, then flow cytometry could be a powerful tool in the prognostication of HF.

### 2.2. Endothelial Progenitor Cells in Healthy Subjects and Patients with Heart Failure

In healthy subjects without cardiovascular comorbidities, there is no significant impairment of their vascular endothelial function. The major function of EPCs is to restore the dysfunctional and inflammatory endothelium, especially in situations including heart failure and coronary syndromes, where there is rupture of the endothelial barrier. Therefore, in healthy people, the number of EPCs is low in peripheral blood and correlates with the low number of circulating vessel wall-derived endothelial cells [57]. They are also inversely associated with age [58]. EPCs constitute 1–5% of the total bone marrow cells, while their population corresponds to 0.0001–0.002% of total peripheral blood mononuclear cells [59].

During the last two decades, the interest in research in the field of EPCs demonstrates an exponential increase by the medical community. Previous studies showed that the number of EPCs reflects vascular repair and, thus, a reduced number of circulating EPCs could predict the occurrence of cardiovascular events [60,61]. Moreover, EPCs could be strongly and independently predictive of mortality in patients with cardiovascular comorbidities and may help to identify patients at increased cardiovascular risk [62,63]. The circulating number of EPCs is lower in patients with cardiovascular comorbidities including diabetes mellitus, hypertension, and hypercholesterolemia [64,65]. The severity of the cardiovascular disease is another important factor that defines EPCs baseline number in circulation. When the severity increases, the number of EPCs and CECs gradually decreases as a result of the endothelial dysfunction in these patients [66,67]. Vascular endothelial function in HF has a strong relation with the number and activity of EPCs and CECs [68]. A decrease in EPCs number suggests a decline in the endothelial repair ability [68]. Heart failure is characterized by reduced bioavailability of NO, systematic inflammation and increased oxidative stress [8,9,10,69]. Therefore, mobilization of EPCs from the bone marrow is being affected. This continuous lack of repair in vascular structure and function causes further deterioration of the endothelial function and progress of the heart failure. Baseline levels of circulating EPCs are low in HF and seem to be similar among patients with preserved and reduced ejection fraction [70]. In other words, severity of HF does not seem to define EPCs number. A possible explanation could be the already established extended vascular inflammation in HF, no matter of the myocardium’s function, and the increased number of inflammatory factors. EPCs are also shown to be significantly lower in HF compared to subjects without HF but with other cardiovascular risk factors, as well as to healthy subjects without cardiovascular risk factors [21,70]. The extended vascular inflammation in HF and the atherosclerotic process that leads to suppressed response in mobilization of progenitor cells from the bone marrow could explain these findings. This reduction in EPCs number and function in HF patients is independently associated with structural abnormalities including increased left atrium diameter, contributes to dysfunctional ventricular remodeling, impaired endothelial repair capacity and is associated with adverse outcomes such as rehospitalizations, cardiac transplantation and sudden cardiac death [71,72]. The relationship between EPCs number and outcomes in chronic HF are demonstrated in Table 1.

## 3. Effects of Exercise on Circulating Endothelial and Progenitor Cells in Heart Failure

### 3.1. Acute Exercise

Acute exercise has been shown to increase EPCs and/or CECs in healthy volunteers [77,78], patients with cardiovascular diseases [79,80] and risk factors [81,82,83], and patients with chronic HF [84,85,86,87,88,89]. There are variables such as intensity of exercise, duration, patient’s medical history, and subgroup of endothelial population defined that determine the acute mobilization and the increase of EPCs number in circulation.

In a previous study held in our Institute, Kourek C. et al. [84] evaluated the effect of acute exercise in 49 consecutive patients with stable chronic HF and a reduced or mid-ranged EF. Most specifically, all patients underwent a ramp incremental symptom-limited maximal cardiopulmonary exercise testing (CPET) on a cycle ergometer and five endothelial cellular populations were identified and quantified by flow cytometry; three subgroups of EPCs (CD34+/CD45−/CD133+, CD34+/CD45−/CD133+/VEGFR-2+, and CD34+/CD133+/VEGFR-2+) and two subgroups of CECs (CD34+/CD45−/CD133− and CD34+/CD45-/CD133-/VEGFR-2). All EPCs and CECs subgroups increased statistically significantly after a single bout of maximal exercise [84]. The same results were repeated a few months later by the same Institution in 44 chronic HF patients following similar methodology [85]. Interestingly, in a post-hoc analysis of the previous study [86], it was shown that exercise-mediated EPCs and CECs mobilization was not associated with the severity of HF (based on cardiopulmonary exercise testing and echocardiographic indices) [86].

The acute effect of a single exercise bout on EPCs in patients with HF was also examined previously by another Institute in Belgium. Specifically, Van Craenenbroeck E.M. et al. [87] performed a symptom-limited CPET in 41 sedentary chronic HF patients on a graded bicycle ergometer and identified two EPCs subgroups defined as CD34+/KDR+/CD32− and CD34+/CD32− progenitor cells via flow cytometry. Patients were divided into two groups of HF severity according to NT-proBNP levels: the group of mild and the group of severe chronic HF. There was also a group of 13 healthy volunteers as control group. They found that CD34+/KDR+/CD32− and CD34+/CD32− cell numbers remained unchanged after a single bout of maximal exercise. However, there was a potent stimulus to reverse circulating angiogenic cells dysfunction by improving their migration in severe (+52%, *p* < 0.05) and mild chronic HF (+31%, *p* < 0.05) and restoring it to levels similar to controls [87]. The same investigators tried to investigate whether the absent immediate effect of acute exercise on EPCs is due to attenuation or delayed mobilization in chronic HF [88]. In HF patients, the initial increase of EPCs was smaller and returned faster to baseline compared to healthy controls. They concluded that the immediate effect of acute exercise on EPCs numbers is not delayed, but significantly attenuated in CHF patients compared to healthy subjects [88]. Effects of acute exercise on EPCs in patients with HF are demonstrated in Table 2.

### 3.2. Exercise Training

The effect of exercise training on EPCs and CECs has been previously assessed in patients with chronic HF. During the last two decades many researchers have investigated the impact of a multi-session exercise training program on EPCs in HF [85,90,91,92,93,94,95,96,97]. The first investigators who evaluated the effect of a regular aerobic exercise training program were Sarto P. et al. [90]. They performed an 8-week supervised aerobic training program in 22 patients with stable chronic HF and evaluated the number of EPCs at the beginning of the study, after 8 weeks of the supervised training program and 8 weeks of the subsequent discontinued supervised aerobic training phase. EPCs were defined as CD34+/KDR+ circulating cells. They also measured plasma concentration of VEGF and stromal-derived factor 1 (SDF-1). Levels of EPCs, VEGF, and SDF-1 increased statistically significantly after the exercise training program but returned to the baseline levels after the discontinuation phase [90]. A couple of years later, Erbs S. et al. [91] enrolled 37 patients with chronic HF either into a 12-week exercise training program or sedentary lifestyle as control group. They defined EPCs as CD34+/KDR+ cells and quantified them by flow cytometry. EPCs were increased in patients who performed exercise training compared to controls [91]. Additional parameters including flow-mediated dilation, skeletal muscle neovascularization, and LV function were also improved after the aerobic training program. This data was confirmed the same year by Van Craenenbroeck E.M. et al. [92] who investigated the impact of exercise training on circulating angiogenic cells function and number of CD34+ and CD34+/KDR+ EPCs in 21 patients with chronic HF. These patients underwent 6-month exercise training and were compared to a non-trained control group of 17 patients and 10 healthy age-matched subjects. Authors showed that exercise training reversed circulating angiogenic cells dysfunction by increasing their migration by 77% and also increased the number of CD34+ and CD34+/KDR+ EPCs in chronic HF [92]. In the contrary, there were no differences in the control group and healthy subjects.

The following years, six more studies were performed in HF patients. Gatta L. et al. [93] evaluated the effect of a 3-week exercise training program on CD34/KDR+ EPCs, MMPs, TIMP-1, and TNF-α in 14 chronic HF patients. Number of circulating CD34+/KDR+ EPCs, as well as MMP-2/TIMP-1 and MMP-9/TIMP-1 ratios increased after exercise training, while a decrease in serum concentration of MMP-1 and TIMP-1 was also observed, indicating their potential role in vascular remodeling [93]. Eleuteri E. et al. [94] performed five sessions of 30-min cycle ergometry (60 rev/min) per week, for 3 months, in 11 chronic HF patients and assessed EPCs (defined as CD45dim/CD34+/KDR+ cells), angiogenetic markers including angiogenin, angiopoietin-1 and -2, VEGF, Tie-2 and SDF-1a, and inflammatory markers including IL-6 and CRP in comparison with ten non-trained HF patients. After the 3-month program, EPCs and angiopoietin-serum levels significantly increased in the HF patients compared to the non-exercised group [94]. Mezzani A. et al. [95] presented similar results with the previous investigators, showing that a 3-month light-to-moderate-intensity aerobic exercise training program of five sessions a week of 30-min cycling (60 rpm) increased significantly the EPCs number (identified as CD45dim/CD34+/KDR+ cells) in trained patients, reaching values similar to those of normal subjects, whereas it remained unchanged in control patients [95].

Interestingly, a few years later, Sandri M. et al. [96] assessed whether disease and aging have additive effects on EPCs or whether beneficial effects of exercise training are diminished in old age. Sixty patients with stable chronic HF and 60 referent controls were randomized either to a training or a control group and exercised four times daily at 60% to 70% of max VO2 under supervision for a month [96]. CD34+/KDR+ EPCs and CD133+/KDR+ EPCs were quantified by flow cytometry and factors such as VEGF, SDF-1, soluble intercellular adhesion molecule (sICAM-1), soluble vascular cell adhesion molecule (sVCAM-1), and asymmetric dimethylarginine (ADMA) were also measured by highly sensitive ELISA [96]. The authors found that the EPCs function improved significantly by 24% in older referent controls above 65 years, while it remained unchanged in young training referent controls below 55 years and controls respectively [96]. Moreover, in both young and older patients with chronic HF, 4 weeks of exercise training resulted in a significant improvement in EPCs numbers and EPCs function (young: number +66% function +43%; *p* < 0.05; older: number +69% function +36%; *p* < 0.05), highlighting the benefits of rehabilitation in HF patients of older age [96]. In a recent study from our Institute, Kourek C. et al. [85] provided further scientific knowledge investigating the effects of different exercise training regimens on EPCs and CECs. Specifically, 44 patients with stable chronic HF were randomized in either a high intensity interval training (HIIT) or a HIIT combined with muscle strength (COM) program and underwent 36 sessions of exercise training [85]. All patients underwent maximum CPET before and after the rehabilitation program and five endothelial populations were quantified by flow cytometry: CD34+/CD45−/CD133+, CD34+/CD45−/CD133+/VEGFR-2+, CD34+/CD133+/VEGFR-2+ (EPCs subgroups) and CD34+/CD45−/CD133−, CD34+/CD45−/CD133−/VEGFR-2+ (CECs subgroups) [85]. Authors demonstrated that all EPCs and CECs populations increased after the program (*p* < 0.01) while there were no differences between HIIT and COM groups. The beneficial effects of both aerobic and muscle strength protocols were similar for all patients, independently of HF severity [85]. Functional capacity assessed by peak VO2 and angiogenetic markers such as VEGF were also improved after the training program [85]. In another interesting study, Chen J. et al. [97] evaluated the effects of exercise training on cardiac function, B-natriuretic peptide (BNP) levels, cell viability, proliferation, apoptosis, and invasion ability of EPCs, eNOS, and VEGF in 80 elderly patients with chronic HF. The training group performed cardiac exercise rehabilitation for 12 weeks, 3–5 times a week while the control group only performed simple exercises at the bedside or indoors and walked freely for 30–60 min per day [97]. Through their results, it was shown that exercise training improved myocardial function and promoted angiogenesis and endothelial function via the improvement of the vitality, proliferation, and invasion of peripheral blood EPCs, and the expression of eNOS and VEGF through the upregulation of the PI3K/AKT pathway [97].

All studies come in agreement that exercise training has beneficial effects on EPCs and CECs by increasing their number in circulation and improving their functional possibilities in patients with HF (Table 2).

## 4. Physiology of Exercise on Circulating Endothelial and Progenitor Cells in Heart Failure

**Figure 3:**
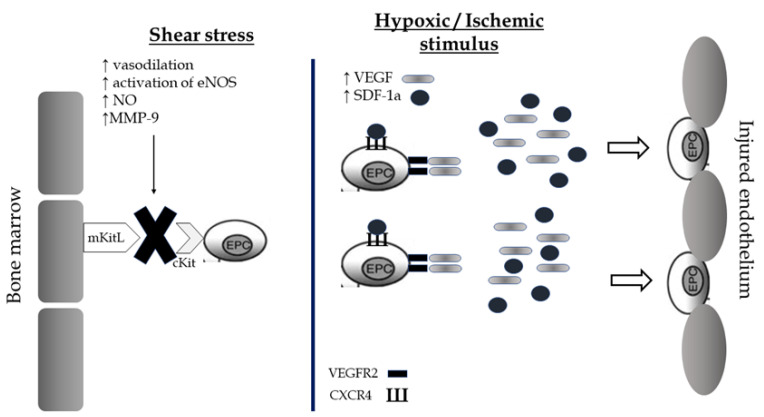
Shear stress and hypoxic/ischemic stimulus as potential mechanisms of mobilization of Endothelial Progenitor cells from the bone marrow and restoration of the endothelial barrier after exercise. EPCs, endothelial progenitor cells; NOS, nitric oxide synthase; NO, nitric oxide; VEGF, vascular endothelial growth factor; SDF, Stromal cell-derived factor; VEGFR, vascular endothelial growth factor receptor; CXCR, C-X-C chemokine receptor.

EPCs are a subtype of immature cells produced in the bone marrow and located between a large number of hematopoietic stem cells and bone marrow stromal cells. These conditions create an appropriate microenvironment which helps them to differentiate into different subsets of cells, mainly into mature endothelial cells. The circulating number of EPCs is low in normal conditions and consists of approximately 0.01% of monocytes. However, there are environmental or physiological factors including estrogens, statins, physical exercise, acute ischemia, and hypoxia that present a direct effect on these cellular populations by stimulating their mobilization from the bone marrow and their differentiation rates into mature endothelial cells. There are two main mechanisms of EPCs mobilization: shear stress and the hypoxic/ischemic stimulus [98]. The combination of both leads to the release of EPCs in circulation promoting their repairment properties on vascular endothelium’s barrier (Figure 3).

During exercise, molecules including the cytokines of granulocyte colony-stimulating factor (G-CSF), matrix metalloproteinases-9 (MMP-9), VEGF, SDF-1a, eNOS, and NO are being activated and promote the release of EPCs from the bone marrow through endothelial sinusoid into circulation [98,99,100]. Most specifically, shear stress, which is caused by exercise, increases endothelium-dependent vasodilation, eNOS activity, and availability of NO [98,99,100]. The increase in the availability of NO leads to the activation of MMP-9. Subsequently, the cleavage of membrane-bound Kit ligand (mKitL) and the binding of sKitL to its receptor on progenitor cells (cKit) contributes to the migration of the EPC to the vascular zone of the bone marrow [98]. The unbonded progenitor cells, which are ready to enter circulation, bring receptors VEGFR2 and C-X-C chemokine receptor type 4 (CXCR4) on their surface. Summing up, the upregulation of the shear stress contributes to the enhancement of endothelialization ability of EPCs. The second pathophysiological mechanism of EPCs mobilization is the hypoxic/ischemic stimulus. Acute exercise causes transient ischemia in the vascular endothelium and the expression of angiogenic factors by hypoxic tissues, including VEGF and SDF-1a, is being increased [98]. These factors bind respectively to the receptors VEGFR-2 and CXCR4 of the progenitor cells and guide them directly to the damaged vascular wall [98]. Thus, the endothelial cell repairs the fractured vessel wall, either as progenitor cell or through its transformation into a mature circulating endothelial cell.

Physiology of exercise and its effects on EPCs is not the same between healthy subjects and patients with heart failure. Healthy subjects usually do not present with inflammation or endothelial dysfunction and, therefore the mobilization of EPCs is much lower or unchanged. The explanation is that the main function of EPCs, which is to restore the injured endothelium, is not useful in a healthy person without systemic inflammation.

Angiogenesis is another significant property of EPCs. Proliferation and migration of EPCs remodels and refines the initial vascular plexus in order to form new vessels [101]. The tight formation between EPCs forms the inner lumen of blood vessels [102], controlling thus paracellular permeability [103], exchange of molecules, and stability of intravascular environment [104]. Authors should discuss the results and how they can be interpreted from the perspective of previous studies and of the working hypotheses. The findings and their implications should be discussed in the broadest context possible. Future research directions may also be highlighted.

## 5. Future Perspectives and Limitations

EPCs are important therapeutic targets and one of the most promising fields in cardiovascular diseases, especially heart failure. Individualized exercise training protocols for the mobilization of endothelial cellular populations could result in LV remodeling, improvement in microcirculation, and increased values of hemodynamic parameters. Moreover, an international network of rehabilitation centers for patients with HF should be created, where specific individualized exercise training protocols would be performed, and reference centers for EPCs identification and quantification should be established in each country. The next step would be to isolate these specific regenerative endothelial populations from the peripheral blood, incubate them in colonies and multiply them in order to inject them back in circulation to adverse structural and functional abnormalities of the myocardium and the vascular endothelial system. Intravenous administration of EPCs in patients with cardiovascular diseases has been proven to be safe and feasible so far, while improving exercise capacity and left ventricular function after a 6-month follow-up [105]; however, its potential beneficial effects remain to be further demonstrated. Another potential application of EPCs is the endothelial progenitor cell capture stent, a stainless-steel stent with the surface of EPCs antibody which could repair the damaged arterial endothelium or form a special blood vessel [106]. Specifically for HF, regenerative medicine via infusion of EPCs showed improvement in LVEF, lower mortality and rehospitalization rates during follow-up, and significant benefits in reduction of infarct size, LV function, functional capacity, and quality of life [107,108]. Development of medications that would stabilize the eNOS mRNA, improve NO bioavailability, and promote protective anti-inflammatory effects to the endothelium [109] would be an extra boost for the mobilization of EPCs after exercise. Thus, further studies investigating the effects of both medication and exercise training in HF are necessary.

Beyond all the future perspectives, there are some common limitations in the international literature for all studies that should be taken into consideration. The lack of a widely approved method of EPCs identification is one of them. In the studies which were included in our review, there were many EPCs populations, defined in various ways with multiple combinations of monoclonal antibodies, that could not allow us to compare differences in EPCs mobilization among these endothelial subgroups and extract safe conclusions. Moreover, the gap in knowledge regarding the potential pathways of mobilization, the role of each endothelial cellular population and its relationship with the clinical status of HF patients is another important limitation that should be referred.

## 6. Conclusions

The present review points out the need for the establishment of a widely approved method of identification of EPCs and CECs and a more clarified definition of the role of each endothelial population subgroup. Acute exercise and exercise training induce EPCs and CECs mobilization, demonstrating its pleiotropic beneficial effects in patients with chronic HFrEF or HFmrEF. EPCs could be a promising field for treatment of cardiovascular diseases and HF. However, further studies are still required to demonstrate the potential pathways and mechanisms of EPCs mobilization.

## Figures and Tables

**Table 1:** Studies investigating the relation between levels of Endothelial Progenitor cells in peripheral circulation and outcomes in chronic heart failure.

Study	Sample Size	EPCs Phenotypes	Primary Outcomes	Results
Koller et al. [73]	185 chronic HF (87 ischemic; 98 non-ischemic)	CD34^+^/CD45^dim^/KDR^+^	All-cause mortality and combined cardiovascular endpoint (death due to cardiovascular events and heart transplantation)	Inverse correlation between EPCs and all-cause mortality. No difference in predictive value between ischemic and non-ischemic chronic HF.
Tahhan et al. [71]	1467 subjects (514 chronic HF; 953 controls)	CD34^+^ CD34^+^/CD133^+^ CD34^+^/VEGFR-2^+^ CD34^+^/CXCR4^+^	Adverse cardiovascular outcomes: - cardiovascular death - hospitalization for HF	3 out of 4 EPCs populations inversely related to rates of all-cause and cardiovascular death. No correlation between EPCs levels and hospitalization.
Berezin et al. [74]	388 chronic HF	CD14^+^/CD309^+^ CD14^+^/CD309^+^/Tie-2+	Utility of biomarkers in assessment of 3-year fatal and non-fatal cardio-vascular events	CD14^+^/CD309^+^/Tie-2+ independently predicted cumulative cardiovascular events in chronic HF patients.
Michowitz et al. [75]	107 chronic HF (ischemic and non-ischemic)	CD31^+^/Tie-2^+^	Relationship between circulating EPCs levels and chronic HF outcomes: - all-cause mortality - hospital admissions	EPCs independently predicted HF mortality. No correlation with hospitalizations due to chronic HF.
Chiang et al. [72]	153 subjects [84 chronic HF (44 HFpEF patients and 40 HFrEF patients) and 69 controls]	CD34^+^/CD45^low^ CD34^+^/KDR^+^/CD45^low^ CD34^+^/KDR^+^/CD133^+^/CD45 ^low^	Relationship between EPCs levels, HFpEF and HFrEF: hs CRP, LVEF, left atrium diameter and the ratio of medial early filling to early diastolic mitral annular velocity.	Decreased circulating EPC numbers in HFpEF and HFrEF patients indicates impaired endothelial turnover.
Kissel et al. [76]	62 subjects [45 chronic HF (25 ischemic and 20 dilated cardiomyopathy) and 17 controls]	CD34^+^/CD45^+^	Relationship between EPCs levels and LV remoddeling process.	Selective impairment of EPCs function in ischemic cardiomyopathy contributes to an unfavorable LV remodeling process.

EPCs, endothelial progenitor cells; HF, heart failure; VEGFR, vascular endothelial growth factor; HFpEF, heart failure with preserved ejection fraction; HFrEF, heart failure with reduced ejection fraction; LVEF, left ventricular ejection fraction; LV, left ventricular; hs-CRP, high sensitivity C-reactive protein.

**Table 2:** Studies investigating the acute and long-term effects of exercise on EPCs and CECs in patients with chronic HF.

Study	Type of Exercise	Study Design	Exercise Prescription	EPCs Phenotypes	Time Points of Blood Samples	Results
Van Craenenbroeck E.M. et al. [89]	Acute	35 sedentary men with chronic HF with EF ≤ 45% (2 groups; Type D and non-Type D patients). - Comparison between Type D and non-Type D personality patients.	Symptom-limited CPET on a graded bicycle ergometer	CD34^+^/KDR^+^	2 time points: Immediately before and 10 min after peak exercise (CPET)	- Circulating EPCs number was reduced by 54% in Type D compared with non-Type D patients. - 60% increase in EPCs in Type D patients. EPCs number remained unchanged in the non-Type D group. - No difference in baseline migratory capacity between groups.
Van Craenenbroeck E.M. et al. [87]	Acute	41 chronic HF patients with EF ≤ 40% (2 groups; 22 mild HF and 19 severe HF) 13 healthy subjects - Comparison of CAC migration and EPCs number between mild and severe HF patients and between HF and healthy subjects	Symptom-limited CPET on a graded bicycle ergometer	CD34^+^/CD3^−^ CD34^+^/KDR^+^/CD3^−^	2 time points: Immediately before and 10 min after peak exercise (CPET)	- CAC migration and CD34^+^ cell numbers were significantly reduced in chronic HF, whereas CD34^+^/KDR^+^ cells were not different from controls. - CPET improved CAC migration in severe (+52%, *p* < 0.05) and mild CHF (+31%, *p* < 0.05), restoring it to levels similar to controls. - No difference in EPCs number after CPET in all groups (*p* > 0.05).
Van Craenenbroeck E.M. et al. [88]	Acute	7 chronic HF patients with EF ≤ 40% and 8 healthy subjects (HS: 4 young and 4 age-matched subjects)	Symptom-limited graded exercise testing (GXT) on a graded bicycle ergometer	CD34^+^/KDR^+^/CD3^−^ CD34^+^/CD3^−^	2 time points: Immediately before and subsequently 10, 30, and 60 min, 2, 4, 8, 12, 24 and 48 h after peak exercise (GXT)	- In both HS groups, CD34^+^/KDR^+^/CD3^-^ EPCs number increased within 10 min following GXT and remained elevated for up to 2 h. - In HF patients, the initial increase was small and normalized within 30 min. - Evolution of CD34^+^/KDR^+^ EPCs numbers over time following GXT overall was attenuated in HF versus HS (*p* = 0.036). - Acute effect of exercise on EPCs number significantly attenuated in chronic HF.
Kourek C. et al. [84]	Acute	49 consecutive patients with stable chronic HF and EF ≤ 49%	Ramp incremental symptom-limited maximal CPET on a cycle ergometer	EPCs (3 subgroups) CD34^+^/CD45^−^/CD133^+^ CD34^+^/CD45^−^/CD133^+^/VEGFR-2^+^ CD34^+^/CD133^+^/VEGFR-2^+^ CECs (2 subgroups) CD34^+^/CD45^−^/CD133^−^ CD34^+^/CD45^−^/CD133^−^/VEGFR-2^+^	2 time points: Immediately before and within 10 min after peak exercise (CPET)	Increase in the mobilizations in all EPCs and CECs populations after maximal exercise (*p* < 0.01).
Kourek C. et al. [86]	Acute	49 consecutive patients with stable chronic HF and EF ≤ 49% [2 groups of HF severity each time according to the median value of peak VO2, predicted peak VO2, VE/VCO2 slope and EF (reduced and mid-ranged)] - Comparison between HF patients of low and high severity.	Ramp incremental symptom-limited maximal CPET on a cycle ergometer	EPCs (3 subgroups) CD34^+^/CD45^−^/ CD133^+^ CD34^+^/CD45^−^/CD133^+^/VEGFR-2^+^ CD34^+^/CD133^+^/VEGFR-2^+^ CECs (2 subgroups) CD34^+^/CD45^−^/CD133^−^ CD34^+^/CD45^−^/CD133^−^/VEGFR-2^+^	2 time points: Immediately before and within 10 min after peak exercise (CPET)	- Statistically significant increase in the mobilization of at least 4 out of 5 cellular populations within lower and higher HF severity group for each severity index after maximal exercise (*p* < 0.05). - No statistically significant differences in the mobilization of EPCs and CECs between severity groups in each comparison (*p* > 0.05).
Kourek C. et al. [85]	Acute	44 patients with stable chronic HF and EF ≤ 49%	Ramp incremental symptom-limited maximal CPET on a cycle ergometer	EPCs (3 subgroups) CD34^+^/CD45^−^/CD133^+^ CD34^+^/CD45^−^/CD133^+^/VEGFR-2^+^ CD34^+^/CD133^+^/VEGFR-2^+^ CECs (2 subgroups) CD34^+^/CD45^−^/CD133^−^ CD34^+^/CD45^−^/CD133^−^/VEGFR-2^+^	2 time points: Immediately before and within 10 min after peak exercise (CPET)	Increase in the mobilizations in all EPCs and CECs populations after maximal exercise (*p* < 0.01).
Sarto P. et al. [90]	Exercise training	22 stable patients with symptomatic chronic HF with EF ≤ 40% and peak VO2 ≤ 25 mL/kg/min. - 8 weeks of supervised aerobic training (SAT) and 8 weeks of subsequent discontinued SAT.	- Incremental upright CPET on a cycle ergometer 3 times per week for 8 weeks. - Each session lasted 55 min, beginning with a 5-min warm-up at 15 Watts followed by 45 min of cycling at the target heart rate and by a 5-min cool-down at 15 Watts.	CD34^+^/KDR^+^	3 time points: At baseline and after 8 weeks of SAT. At least 48 h after the last exercise session.	- Levels of EPCs increased (*p* < 0.001 vs. baseline) but returned to the baseline levels after discontinued SAT. - Similar results for VEGF/SDF-1 (*p* < 0.001 vs. baseline). - Increase in peak VO2, exercise duration, anaerobic threshold, exercise capacity and EF, and improvement in NYHA class after 8 weeks of SAT.
Erbs S. et al. [91]	Exercise training	37 patients with chronic HF and EF ≤ 30% [2 groups; exercise training (ET) group and control group]. - Evaluation of the effect of exercise training on EPCs and other indices.	ET group: In-hospital during the first 3 weeks, exercise 3 to 6 times daily for 5 to 20 min on a bicycle ergometer at 50% of peak VO2. Then on discharge, 20 to 30 min for 12 weeks at home and 60 min of supervised exercise each week consisting of walking, calisthenics and noncompetitive ball games. Control group: 12 weeks sedentary life	CD34^+^/KDR^+^	2 time points: At the beginning of the study and after 12 weeks	ET improved: - Number of EPCs by +83 ± 60 vs. −6 ± 109 cells/mL in controls (*p* = 0.014). - EPCs migratory capacity by +224 ± 263 vs. −12 ± 159 EPCs/1000 plated EPCs in controls (*p* = 0.03) - VO2 max by +2.7 ± 2.2 vs. −0.8 ± 3.1 mL/min/kg in controls (*p* = 0.009) - EF by +9.4 ± 6.1 vs. −0.8 ± 5.2% in controls (*p* < 0.001) - Flow-mediated dilation by +7.43 ± 2.28 vs. +0.09 ± 2.18% in controls (*p* < 0.001) - Skeletal muscle capillary density by +0.22 ± 0.10 vs. −0.02 ± 0.16 capillaries per fiber in controls (*p* < 0.001).
Van Craenenbroeck E.M. et al. [92]	Exercise training	21 sedentary chronic HF patients with EF ≤ 40% underwent 6-month exercise training and were compared to a sedentary control group (*n* = 17) and 10 healthy age-matched subjects. - Evaluation of the impact of exercise training on CAC function and number of EPCs in patients with chronic HF. - Evaluation of the effect of acute exercise on CAC and EPCs in sedentary and trained patients.	60 min per session, 3 times/week for 6 months. Endurance training intensity: 90% of heart rate CXT: CPET on a graded bicycle ergometer with exercise load at 20 or 40 W, with incremental steps of 10 or 20 W/min.	CD34^+^/KDR^+^/CD3^−^ CD34^+^/CD3^−^	4 time points: Before and 10 min after peak exercise (GXT) at baseline and after 6 months	- 77% increase in CAC migration (*p* = 0.001) after 6 months of exercise training. - The GXT-induced improvement at baseline was no longer observed after training. - ^−^ Number of CD34^+^/KDR^+^/CD3^−^ EPCs increased after 6 months (*p* = 0.021), but was not affected by GXT.
Gatta L. et al. [93]	Exercise training	Training group: 14 patients with chronic HF due to coronary artery disease with EF < 40% Control group: 15 matched patients with chronic HF and EF 55 ± 3% (only baseline measurements) - Evaluation of the effect of exercise training on EPCs and other indices.	Training group: 2 daily sessions for 6 days a week for 3 weeks. Session: calisthenics, 30 min of aerobic exercise on a bicycle ergometer with incremental, workload. Intensity at 85% of HRmax, or at 75% of HRmax for >65 years old. Initial CPET on an electrically braked bicycle ergometer (1 min of unloading pedaling and increased by 10 W every minute until pedaling rate <60 rpm).	CD34/KDR^+^	2 time points: At admission and at least 24 h after the last exercise session.	After exercise training: - 6MWT increased from 154 ± 27 to 233 ± 48 m (*p* < 0.0001) - Number of EPCs increased from 5 ± 3 to 9 ± 6 cells/mL (*p* < 0.05) - MMP-1 and TIMP-1 decreased from 11.4 ± 2.4 to 6.3 ± 1.1 ng/mL, and from 320.4 ± 41.2 to 167.2 ± 12.6 ng/mL, respectively (*p* < 0.01) - MMP2/TIMP-1 and MMP-9/TIMP-1 ratios increased. - Increased CFU-EC proliferation in cultures performed with serum. - IL-1β, IL-6, MMP-2, MMP-9 remained unchanged after training (*p* > 0.05)
Eleuteri E. et al. [94]	Exercise training	21 male patients with chronic HF and EF ≤ 40% were randomized in either a 3-month aerobic training (CHF-TR) performed at home, or control group (CHF-C). - Evaluation of the effect of exercise training on EPCs, angiogenesis and inflammation compared to controls.	CHF-TR: 5 sessions a week of 30-min cycle ergometry (60 rev/min) at a power and heart rate corresponding to VAT, preceded and followed by a 5-min warm-up and cool-down unloaded period. Controls: normal lifestyle activities.	CD45^dim^/CD34^+^/KDR^+^	2 time points: At baseline before and after the 3-month exercise training program.	- EPCs count and AP-2 serum levels significantly increased in the CHF-TR group after exercise training program compared to CHF-C where it remained unchanged. - Peak VO2 and VAT VO2 improved significantly by 9% (*p* = 0.01) and 14% (*p* = 0.009), respectively in the CHF-TR, but not in the CHF-C group. - Significant improvement in endothelial-mediated vasodilation of the brachial artery in CHF-TR (5.1 ± 0.7% to 7.0 ± 0.5%, *p* = 0.03) but not in CHF-C group.
Mezzani A. et al. [95]	Exercise training	30 chronic HF patients with EF ≤ 40% were randomized to 3 months of light-to-moderate-intensity AET (CHF-AET) or control (CHF-C or normal volunteers). - Evaluation of adaptations of pulmonary VO2 on-kinetics in response to a 3-month light-to-moderate-intensity AET program in HF.	CHF-AET: 5 sessions a week of 30-min cycling (60 rpm) for 3 months followed by 5-min warm-up and cool-down periods of unloaded cycling. An incremental CPET was repeated 6 weeks after protocol start to adjust training stimulus intensity. CHF-C: daily lifestyle and activities without undergoing any formal training protocol.	CD45^dim^/CD34^+^/KDR^+^	2 time points: At baseline and after the end of the exercise training program	After exercise training: - phase I duration, phase II τ, and MRT were significantly reduced (−12%, −22%, and −19%, respectively) and peak VO2, peak Δ[deoxy(Hb+Mb)], and EPCs increased (9%, 20%, and 98%, respectively) in CHF-AET, but not in CHF-C. - Peak Δ[deoxy(Hb+Mb)] and EPCs relative increase correlated significantly to that of peak VO2 (*r* = 0.61 and 0.64, respectively, *p* < 0.05)
Sandri M. et al. [96]	Exercise training	60 patients with stable chronic HF with EF ≤ 40% and 60 referent controls (RC) to a training or a control group. In total, 4 groups; RC ≤ 55 years, RC ≥ 65 years, CHF ≤ 55 years, CHF ≥ 65 years. - Assessment whether disease and aging have additive effects on EPCs or whether beneficial effects of exercise training are diminished in old age.	Training group: aerobic exercise 4 times daily for 15–20 min on a bicycle ergometer at 60% to 70% of VO2max for 4 weeks under supervision.	CD34^+^/KDR^+^	2 time points: At baseline and after the 4-week exercise training program	At baseline: - Reduced EPCs number (young: 190 ± 37 CD34/KDR positive cells/mL blood; older: 131 ± 26 CD34/KDR positive cells/mL blood; *p* < 0.05) and function (young: 230 ± 41 migrated cells/1000 plated cells; older: 185 ± 28 cells/1000 plated cells; *p* < 0.05) in older referent controls compared to younger. - Impaired EPCs number (young: 85 ± 21 CD34/KDR positive cells/mL blood; older: 78 ± 20 CD34/KDR positive cells/mL blood) and EPCs function (young: 113 ± 26 cells/1000 plated cells; older: 120 ± 27 cells/1000 plated cells) in both young and older chronic HF patients. After exercise training: - EPCs function improved by 24% in older referent controls (*p* < 0.05), while it remained unchanged in young training referent controls and controls respectively. - Significant improvement in EPCs numbers and EPCs function (young: number +66% function +43%; *p* < 0.05; older: number +69% function +36%; *p* < 0.05) in both young and older patients with HF. - Significant increase in flow mediated dilatation in the training groups of young/older chronic HF patients and in older referent controls.
Kourek C. et al. [85]	Exercise training	44 patients with stable chronic HF with EF ≤49% randomized in either high-intensity interval training (HIIT) or HIIT combined with muscle strength (COM), and subsequently divided in 2 groups according to NYHA status (NYHA II or III). - Assessment of the effect of exercise training on EPCs at rest and acutely. - Evaluation of differences between 2 exercise training protocols and between patients of different NYHA status.	36-session exercise training program, 3 times per week. HIIT: Cycling for 7 min warm-up at 45% peak VO2 on a stationary bike, followed by 3 min at 50% peak VO2. Four 4-min intervals at 80% peak VO2 were alternated with 3-min repetitions at 50% peak VO2. Workload intensity gradually increased to reach + 25% by the end. Total duration of each session 31 min. In the end, narrow corridor walking, backward narrow corridor walking and side walking in both sides. COM: HIIT including strength training of 2–3 sets, 10–12 repetitions, 60–75% of 1RM (knee extension, knee flexion and chest press exercises with 1-min rest between sets)	EPCs (3 subgroups) CD34^+^/CD45^−^/CD133^+^ CD34^+^/CD45^−^/CD133^+^/VEGFR-2^+^ CD34^+^/CD133^+^/VEGFR-2^+^ CECs (2 subgroups) CD34^+^/CD45^−^/CD133^−^ CD34^+^/CD45^−^/CD133^−^/VEGFR-2^+^	4 time points: Immediately before and within 10 min after maximal exercise (CPET), at baseline before and after the exercise training program	- Increase in all EPCs and CECs populations at rest (*p* < 0.01). - Increase in 4 out of 5 endothelial cellular populations in the acute response after CPET after the exercise training program. - Increase in EPCs at rest and the acute response after exercise training within each exercise training group and each NYHA class. - Increase in VEGF and decrease of CRP within each exercise training group and each NYHA class. - No differences in EPCs and CECs number, VEGF and CRP between HIIT and COM or NYHA II and NYHA III groups (*p* > 0.05).
Chen J. et al. [97]	Exercise training	80 elderly patients (between 65 and 80) with chronic HF of grade II or III randomly divided in training and control group. - Evaluation of the effects of exercise training on EPCs in elderly patients with chronic HF.	Training group: exercise training for 12 weeks, 3–5 times a week and free walk for 30–60 min a day. Control group: treated routinely and walked freely for 30–60 min every day, simple exercises at the bedside or indoors.	CD34^+^/KDR^+^	2 time points: At baseline before and immediately after the exercise training program	At baseline: - No significant differences in BNP, EPCs viability, proliferation, apoptosis, and invasion ability, levels of the PI3K/AKT pathway, eNOS and VEGF between the two groups before treatment (*p* > 0.05). After exercise training, the training group compared to the control group showed: - Higher LVEF and LVFS and lower LVEDD and LVESD (*p* < 0.05). - Lower BNP levels (*p* < 0.05). - Higher cell viability, proliferation, invasion ability of EPCs, and levels of PI3K, AKT, eNOS, and VEGF mRNA and protein (*p* < 0.05). - Lower apoptosis rate (*p* < 0.05).

EPCs: Endothelial progenitor cells; CECs: Circulating endothelial cells; HF: Heart failure; EF: Ejection fraction; CPET: Cardiopulmonary exercise testing; CAC: Circulating angiogenic cells; GXT: Graded exercise testing; VEGFR: Vascular endothelial growth factor; 6MWT: Six minute walking test; MMP: Matrix metalloproteinases; TIMP: Tissue inhibitors of metalloproteinases; CFU-EC: Colony forming unit-endothelial cells; IL: Interleukin; CRP: C reactive protein; NYHA: New York Heart Association; HIIT: High intensity interval training; BNP: B-type natriuretic peptide; VAT: Ventilatory anaerobic threshold; PI3K: Phospoinositide 3-kinases; AKT: Serine/threonine kinase; eNOS: Endothelial nitric oxide synthase; LVEF: Left ventricular ejection fraction; LVFS: Left ventricular fractional shortening; LVEDD: Left ventricular end-diastolic diameter; LVESD: Left ventricular end-systolic diameter.
